# Biomonitoring of Exposure to Organophosphate Pesticides: McKelvey et al. Respond

**DOI:** 10.1289/ehp.1408444R

**Published:** 2014-07-01

**Authors:** Wendy McKelvey, J. Bryan Jacobson, Daniel Kass, Dana B. Barr

**Affiliations:** 1 Division of Environmental Health, New York City Department of Health and Mental Hygiene, New York, New York, USA; 2 Emory University, Atlanta, Georgia, USA; E-mail: wmckelve@health.nyc.gov

In their letter, Ross and Ginevan criticize the conclusion of our recent article on pesticide biomonitoring in New York City (NYC), in which we stated that

Estimates of exposure to pyrethroids and dimethyl organophosphates were higher in NYC than in the United States overall, underscoring the importance of considering pest and pesticide burdens in cities when formulating pesticide regulations. (McKelvey et al. 2013)

However, in our article, we raised similar concerns as Ross and Ginevan—namely, the possibility that the exposures we measured could have been exposures to the metabolites themselves or to compounds that originated in food (either as the parent compound or the metabolite). We discussed the reduced likelihood that the dimethyl metabolites originated from structural pest control, based on limited use of these products for indoor pest control in NYC at the time. We also noted the possibility of exposure through use of flea and tick control products on pets or for outdoor garden care. Tetrachlorvinphos is a common ingredient in flea and tick products and a credible source of exposure in New York City, where pets tend to stay indoors and living spaces are smaller. We call attention to recent concern over indoor use of dichlorvos, a dimethyl organophoshate ingredient of insecticidal pest strips, which could have also been a source of exposure (Tsai et al. 2014). Our interpretation of elevated levels of urinary dimethyl phosphates in NYC adults was consistent with the recommendation of Krieger et al. (2012), cited by Ross and Ginevan in their letter, that use of dialkylphosphate compounds as biomarkers of exposure to organophosphates be limited to identifying potentially exposed groups or changing use patterns. We followed this recommendation in reporting evidence for increased exposure to dimethyl organophosphate products at the high end of the distribution in the general NYC adult population.

More importantly, pyrethroids are now the largest share of structural pesticide applications in NYC (New York City Department of Health and Mental Hygiene 2014). The 95th percentiles of urinary pyrethroid metabolites we measured in NYC adults were higher than those in the general U.S. population. Exposure may be greater in NYC because of more applications, drift between housing units in multiunit dwellings, or other factors related to the built environment. The registration and regulation of pesticides do not adequately account for such phenomena. More regulatory oversight of pesticide use in densely populated areas such as NYC is a logical response.
